# Focal Cortical Resection and Hippocampectomy in a Cat With Drug-Resistant Structural Epilepsy

**DOI:** 10.3389/fvets.2021.719455

**Published:** 2021-07-20

**Authors:** Daisuke Hasegawa, Rikako Asada, Yuji Hamamoto, Yoshihiko Yu, Takayuki Kuwabara, Shunta Mizoguchi, James K. Chambers, Kazuyuki Uchida

**Affiliations:** ^1^Laboratory of Veterinary Radiology, Faculty of Veterinary Science, Nippon Veterinary and Life Science University, Musashino, Japan; ^2^The Research Center for Animal Life Science, Nippon Veterinary and Life Science University, Musashino, Japan; ^3^Veterinary Medical Teaching Hospital, Nippon Veterinary and Life Science University, Musashino, Japan; ^4^Laboratory of Veterinary Pathology, Graduate School of Agricultural and Life Sciences, The University of Tokyo, Bunkyo-ku, Japan

**Keywords:** cat, drug-resistant epilepsy, electroencephalography, electrocoricography, epileptogenic zone, epilepsy surgery, magnetic resonance imaging, video-EEG

## Abstract

Epilepsy surgery is a common therapeutic option in humans with drug-resistant epilepsy. However, there are few reports of intracranial epilepsy surgery for naturally occurring epilepsy in veterinary medicine. A 12-year-old neutered male domestic shorthair cat with presumed congenital cortical abnormalities (atrophy) in the right temporo-occipital cortex and hippocampus had been affected with epilepsy from 3 months of age. In addition to recurrent epileptic seizures, the cat exhibited cognitive dysfunction, bilateral blindness, and right forebrain signs. Seizures had been partially controlled (approximately 0.3–0.7 seizures per month) by phenobarbital, zonisamide, diazepam, and gabapentin until 10 years of age; however, they gradually became uncontrollable (approximately 2–3 seizures per month). In order to plan epilepsy surgery, presurgical evaluations including advanced structural magnetic resonance imaging and long-term intracranial video-electroencephalography monitoring were conducted to identify the epileptogenic zone. The epileptogenic zone was suspected in the right atrophied temporo-occipital cortex and hippocampus. Two-step surgery was planned, and a focal cortical resection of that area was performed initially. After the first surgery, seizures were not observed for 2 months, but they then recurred. The second surgery was performed to remove the right atrophic hippocampus and extended area of the right cortex, which showed spikes on intraoperative electrocorticography. After the second operation, although epileptogenic spikes remained in the contralateral occipital lobe, which was suspected as the second epileptogenic focus, seizure frequency decreased to <0.3 seizure per month under treatment with antiseizure drugs at 1.5 years after surgery. There were no apparent complications associated with either operation, although the original neurological signs were unchanged. This is the first exploratory study of intracranial epilepsy surgery for naturally occurring epilepsy, with modern electroclinical and imaging evidence, in veterinary medicine. Along with the spread of advanced diagnostic modalities and neurosurgical devices in veterinary medicine, epilepsy surgery may be an alternative treatment option for drug-resistant epilepsy in cats.

## Introduction

Epilepsy is a common functional cerebral disorder in cats and dogs. At present, treatment of canine and feline epilepsy is almost limited to antiseizure drug (ASD) therapy. Although most feline patients with epilepsy respond to ASD therapy and have a good prognosis, 20–39% of cases do not respond to ASDs and are referred to as drug-resistant epilepsy (DRE), which results in a poor outcome and quality of life (1–4). DRE is estimated to exist in approximately 30% of human and canine epilepsy patients (5, 6).

In human medicine, surgical treatment, so-called “epilepsy surgery,” has been established as a common strategy for patients with DRE (7, 8). In order to plan for epilepsy surgery, various evaluations, so-called “presurgical evaluations,” must be performed to localize the epileptogenic zone (historically known as the “focus”). The epileptogenic zone is conceptually defined as the minimum amount of cortex that must be resected or disconnected to produce seizure freedom, which is considered from the results of presurgical evaluations. Five abnormal zones, that is, symptomatogenic, irritative, seizure-onset, structurally abnormal, and functional deficit zones, are essential to presume the epileptogenic zone, and are investigated by specific modalities (9). Epilepsy surgery includes three methodologies, that is, resection, disconnection, and neuromodulation, such as vagus nerve stimulation and deep brain stimulation. Resection surgery is the most curative method, which involves the direct removal of the epileptogenic zone, and is applied for focal (localized-related) epilepsy. Especially, anterior temporal lobectomy or selective amygdalohippocampectomy is commonly performed for intractable mesial temporal lobe epilepsy (MTLE) with hippocampal sclerosis and shows a good postoperative outcome (10).

A recent survey of research priorities for idiopathic epilepsy in dogs revealed that non-ASD management, including epilepsy surgery, is highly expected by dog owners, general practice veterinarians, and veterinary neurologists (11). However, research on epilepsy surgery in the veterinary field is generally limited to experimental studies, with the majority concerning neuromodulation (12–17). There are some case reports regarding intracranial surgery in canine patients with seizures, but most of those lack modern imaging or electrodiagnostic evidence (18–21).

Feline temporal lobe epilepsy (FTLE), which resembles human MTLE, is the most commonly encountered epilepsy syndrome in cats (3, 22, 23). In fact, previous basic studies in human MTLE had progressed using feline seizure models such as kindling and kainic acid models (22). Under such a background, hippocampectomy, a type of resection surgery, would be naturally expected to be a surgical option for drug-resistant FTLE. Methodologies to detect the epileptogenic zone in FTLE using familial epileptic cats (24–29) and a cadaveric study of feline hippocampectomy (17) have been reported.

Recent technological advances, for example, digital electroencephalography (EEG) with video monitoring and high-field and advanced magnetic resonance imaging (MRI), and practical experiences of canine and feline brain surgery using a surgical microscope, ultrasonic aspirator, and neuronavigator, suggest that presurgical evaluations of the epileptogenic zone and epilepsy surgery could also be performed in veterinary medicine. On the basis of these backgrounds, we report a feline case with structural DRE that underwent intracranial epilepsy surgery with presurgical evaluations and its 1.5-year follow-up.

## Case Presentation

A 12-year-old neutered male domestic shorthair cat had suffered from DRE for at least 2 years.

### History

The cat was rescued by a local veterinarian at approximately 3 months of age. Since recurrent epileptic seizures and bilateral blindness were noticed, the cat was introduced to the authors' laboratory to investigate the underlying cause. At this time (when the cat was 3 months old), neurological examinations revealed walking toward the right (right circling in a large room), subtle hypermetric gait, decreased postural responses on the left side, and a bilateral deficit of the menace response with a normal pupillary light reflex, suggesting a forebrain lesion (more severe in the right hemisphere). Seizure type was focal seizures that consisted of behavioral arrest, star-gazing, facial twitching, mydriasis, salivation, circling, falling, and then (sometimes) evolving to generalized tonic-clonic seizures (GTCS) (Supplementary Video 1). Seizure duration was 30–60 s (focal seizures) to 2–3 min (if evolving to GTCS). General physical examinations, complete blood count, serum chemistry, blood gas, electrolytes, and urinalysis were within the reference intervals, and thoracic and abdominal radiographs were normal. Detection tests for FeLV (antigen), FIV (antibody), FCoV (PCR), and *Toxoplasma gondii* (PCR) were negative. Conventional MRI (1.5 Tesla) including T1-weighted (T1W) with/without contrast enhancement, T2-weighted (T2W), and fluid-attenuated inversion recovery (FLAIR) imaging of the brain revealed atrophy of the temporo-occipital cortex and hippocampus in the right hemisphere with dilation of the ipsilateral lateral ventricle and subtle dilation of the contralateral (left) lateral ventricle. However, the atrophic lesions had no obvious change in signal intensity on each sequence. There was no other structural abnormality or abnormal contrast enhancement in other regions. The results of cerebrospinal fluid analysis were also within the reference range. These clinicopathologic findings led to the diagnosis of structural epilepsy with right hippocampal and temporo-occipital cortical atrophy. As to the cause of the structural abnormalities, a perinatal cerebral vascular accident was suspected; however, it remains a matter of speculation. After diagnosis, the cat was transferred to our laboratory because it was difficult to keep it in an ordinary household.

The cat was accepted into our laboratory as a rare case for treatment and research with ethical approval from the Institutional Animal Care and Use Committee and Animal and Human Biology Research Ethics Committee of the Nippon Veterinary and Life Science University (accession numbers; 27S-60, 28K-6, 29K-2, and 2019K-1). After diagnosis, ASD therapy with phenobarbital was started and maintained (2–4 mg/kg, q12h, PO; serum concentration 19–30.5 μg/ml) for 1 year with 1–2 seizures per 3 months (1–2 sz/3 m). Until the cat was 10 years old, seizure severity and frequency often deteriorated, and ASD therapy was maintained while dosing up and/or adding other drugs including diazepam, zonisamide, and gabapentin to control seizure frequency to <1 seizure per month (sz/m). However, after 10 years of age, the seizures could not be controlled (2–3 sz/m) even using multiple ASDs (finally, phenobarbital 2 mg/kg, q12h; diazepam 0.8 mg/kg, q12h; zonisamide 15 mg/kg, q12h; and gabapentin 15 mg/kg, q8h). The seizures were often clustered and/or developed to status epilepticus, and diazepam (1.0–2.0 mg/kg, IV) and levetiracetam (20 mg/kg, q8h, PO or IV) were also administered at that time. Most seizures were the same type as mentioned above, but sometimes they initiated from bilateral mydriasis and facial clonus (without behavioral arrest or star-gazing) and evolved to GTCS. The ratio of occurrence of this minor seizure type to the main seizure type was approximately 4:1. Furthermore, the cat developed severe cognitive dysfunction. During these years, physical and neurological examinations, blood tests, EEG examinations, and MRI scans were performed 1–2 times per year, but there was no apparent change, except for recurrent cystitis, which was treated adequately. Ovarian hysterectomy was performed at the time of MRI at 1 year of age. Combined vaccinations (Fel-O-Vax^®^ 5; Zoetis Japan, Tokyo, Japan) were administered every 3 years.

When the cat reached the age of 12 years, we decided to perform epilepsy surgery due to the following reasons: (1) the seizures frequently clustered and developed into status epilepticus; (2) further ASDs might not be effective; (3) the cost for medications was increasing constantly; and (4) preparations to perform intracranial EEG and epilepsy surgery were made in our previous studies of epilepsy (24–29), and a video-EEG monitoring system and surgical microscope were installed in our laboratory.

### Presurgical Evaluations

After the decision to perform epilepsy surgery was taken (2 weeks before surgery for intracranial electrode placement), neurological and physiological examinations, complete blood count, serum chemistry, electrocardiography, and indirect blood pressure were reevaluated. There was no change in the neurological findings from those mentioned above and they suggested mainly a right forebrain lesion with bilateral blindness and severe cognitive dysfunction. Blood tests remained within the reference range. There was no specific change on electrocardiography or blood pressure measurement.

**Figure 1:**
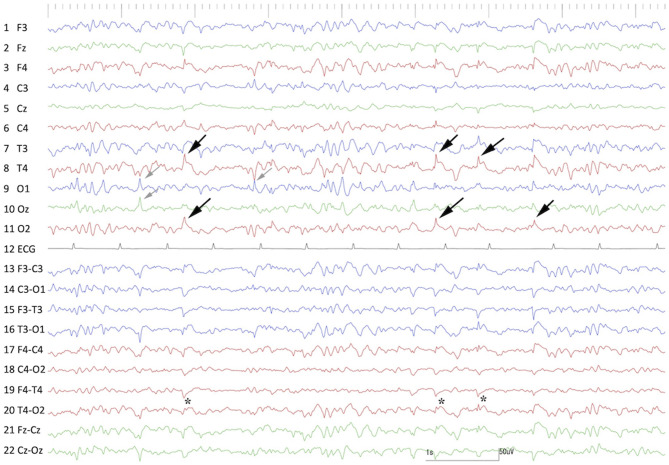
Preoperative interictal scalp EEG under sedation with medetomidine. Remarkable spikes were observed frequently at T4 and O2 (black arrows) with phase reversal (^⋆^) at T4. Sporadic spikes were observed at O1 and Oz (gray arrows). Channels 1–11 were monopolar, and channels 13–22 were bipolar montages. Channel 12 was electrocardiography.

Preoperative scalp EEG (Neurofax EEG-1200; Nihon Kohden, Tokyo, Japan) with subdermal wire electrodes was performed for 30 min under sedation with medetomidine (20 μg/kg, IM). The EEG recording conditions are described in Supplementary Data 1. Isolated spikes or sharp-waves were observed on the right temporal (T4) and occipital (O2) regions dominantly and frequently, but sometimes they were also observed on the left occipital region (O1) (Figure 1).

**Figure 2:**
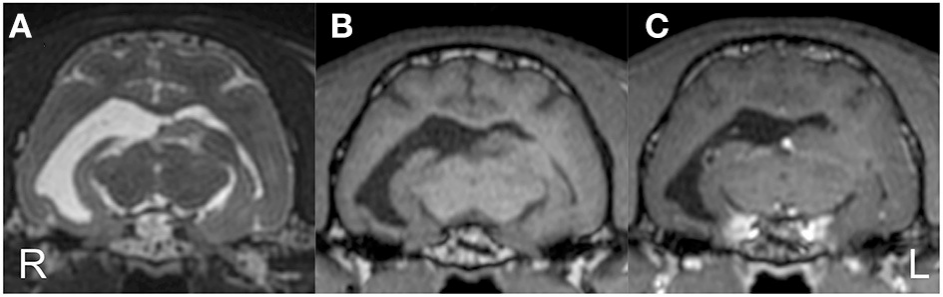
Preoperative structural MRI. Transverse T2-weighted **(A)**, T1-weighted **(B)**, and contrast-enhanced T1-weighted **(C)** images at the level of the medial geniculate body. The right temporo-occipital cortex and hippocampus were markedly atrophied with an extended ipsilateral lateral ventricle. The contralateral (left) ventricle was also slightly dilated.

**Figure 3:**
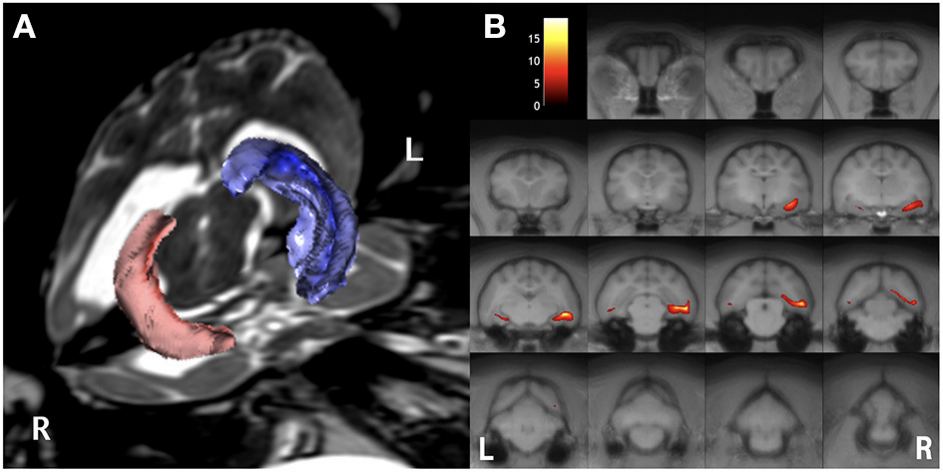
Preoperative imaging analysis of structural MRI. Volume rendering for hippocampal volumetry **(A)** and the result of statistical voxel-based morphometry analysis for gray matter **(B)**. The volume of the right (red) hippocampus was 0.12 cm^3^, whereas the left (blue) was 0.24 cm^3^ **(A)**. On the map of voxel-based morphometry analysis **(B)**, the colored regions indicate significantly different areas compared with 12 healthy controls. Note, the left (L)/right (R) direction of voxel-based morphometry was different from the conventional direction.

**Figure 4:**
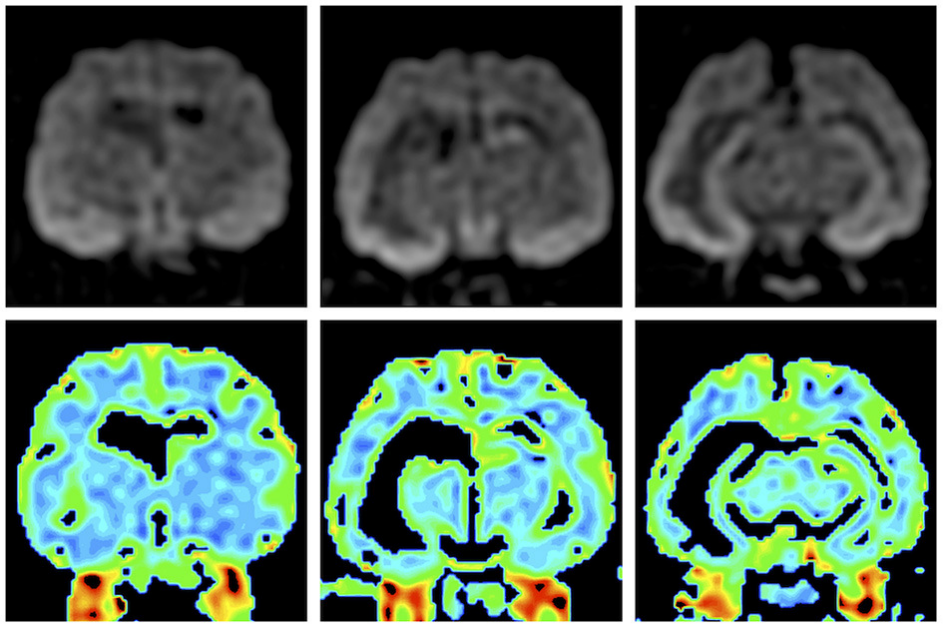
Preoperative isotropic diffusion-weighted imaging (upper row) and colored apparent diffusion coefficient maps (lower row). Bilateral hippocampi and the right amygdala and temporo-occipital cortex showed hyperintensity, but there were no significant changes on the apparent diffusion coefficient map.

Preoperative MRI was performed with a 3.0 T MRI system (Signa HDxt; GE Healthcare, Tokyo, Japan) under general anesthesia, which was induced by propofol and maintained with isoflurane. Obtained images included 3D T1W (spoiled gradient echo with IR preparation) with/without contrast enhancement (IV administration of gadodiamide), 3D T2W (3D T2 Cube), transverse plane FLAIR, T2^*^-weighted (T2^*^W), diffusion-weighted imaging (DWI), diffusion tensor imaging (15 axes), and dynamic susceptibility contrast perfusion MRI (27, 28). The MRI sequences are summarized in Supplementary Data 1. 3D T2W images were used for hippocampal volumetry by the semiautomatic region of interest method (26), and 3D T1W images were also used for statistical analysis using voxel-based morphometry (29). As described above, MRI revealed the right hippocampal and temporo-occipital atrophy without apparent signal changes on T1W, T2W, FLAIR, and contrast-enhanced T1W imaging (Figure 2). The volumes of the right and left hippocampi were measured by 3D volumetry using 3D T2W imaging as 0.119 and 0.24 cm^3^, respectively (reference range of the feline unilateral hippocampus is 0.227 ± 0.02 cm^3^) (Figure 3A) (26). Voxel-based morphometry analysis revealed a significantly decreased volume of the right hippocampus and temporo-occipital cortex (Figure 3B) and increased volume of the right lateral ventricle compared with reference feline brains (29). There was no evidence of hemorrhage on T2^*^W images. Isotropic DWI showed high signal intensity on both hippocampi and the right amygdala and lateral temporo-occipital cortex, but those apparent diffusion coefficient values were not lower than in the other cortex (Figure 4).

On the basis of the results of ictal symptoms, scalp EEG, and structural MRI, we suspected that the epileptogenic zone (symptomatogenic, irritative, and structurally abnormal zones) included, at least, the right atrophic hippocampus and temporo-occipital cortex. To confirm and investigate the “seizure-onset zone,” we performed long-term intracranial EEG (iEEG) monitoring with synchronous video recording, so-called intracranial video-EEG (iVEEG).

#### Preparation for iVEEG Monitoring

**Figure 5:**
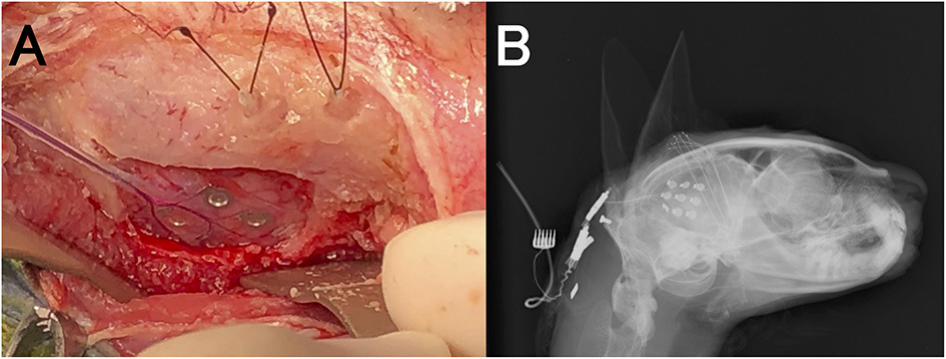
Placement of intracranial electrodes. **(A)** Implantation of a 9-channel ECoG grid (on the dura covering the temporo-occipital cortex) and depth electrodes (into the hippocampus and amygdala). **(B)** Lateral radiograph after implantation of intracranial electrodes.

To place the intracranial electrodes, anesthesia was induced with propofol and maintained with isoflurane and oxygen inhalation. A constant rate of fentanyl infusion (0.1–0.3 μg/kg/min) was administered for analgesia during the operation. Under general anesthesia, the head of the cat was fixed in a stereotaxic frame (Model 1430M; David Kopf Instruments, Tujunga, CA, USA), and computed tomography (Aquilion PRIME; Canon Medical Systems, Tochigi, Japan) and MRI for stereotactic depth electrode insertion were performed. After those scans, the coordinates of the right and left amygdala and ventral portions of the hippocampi were measured, and the cat was moved and set on the operating table. After shaving and sterilization, a U-shaped incision beyond the midline was made on the right scalp and the right temporal muscle and a part of the left temporal muscle were detached from the skull. Small burr holes were made by marking the points on the skull over the extended line of the coordinates for the bilateral amygdala and hippocampus. Order-made epoxy-coated stainless depth bipolar electrodes (0.4 mm diameter and 25 mm length; Unique Medical, Tokyo, Japan) were inserted stereotactically into the bilateral amygdala and hippocampus according to those coordinates (Supplementary Data 1). Craniectomy was performed on the right temporal bone, and the dura on the temporo-occipital area was exposed. We placed order-made ECoG electrodes (1.5 cm × 1.5 cm grid-type silicon-sheet electrodes with 9 exploration electrodes, 5.0 mm interval; Unique Medical) on the dura mater (i.e., epidural space) overlying the atrophic temporo-occipital cortex (Figure 5A) (Supplementary Data 1). A reference screw electrode was placed in the external occipital protuberance and an internal ground was implanted subcutaneously in the back of the neck. The depth and ECoG electrodes were connected to the lead line, which was tunneled subcutaneously to the nape (Figure 5B). The temporal muscle and skin were sutured according to the usual method. The lead was connected to the EEG machine to confirm the connections and the ability for EEG measurement. After that, anesthesia was stopped, and the cat recovered normally. A fentanyl patch (2.5 mg/sheet/head) was used for 3 days, and cefazoline (25 mg/kg, BID) was administered for 14 days after surgery.

#### Long-Term iVEEG Monitoring

From 3 days after electrode implantation surgery, 24-h continuous iVEEG was monitored within an epilepsy monitoring cage (Supplementary Data 1) for 18 days. During this monitoring period, ASDs were limited to phenobarbital and diazepam (i.e., zonisamide and gabapentin were discontinued). Although the cat with the lead line was connected to the EEG machine via a slip-ring connector, it could move freely in the monitoring cage. Recorded iVEEG data were stored in the internal hard disk of the EEG machine.

**Figure 6:**
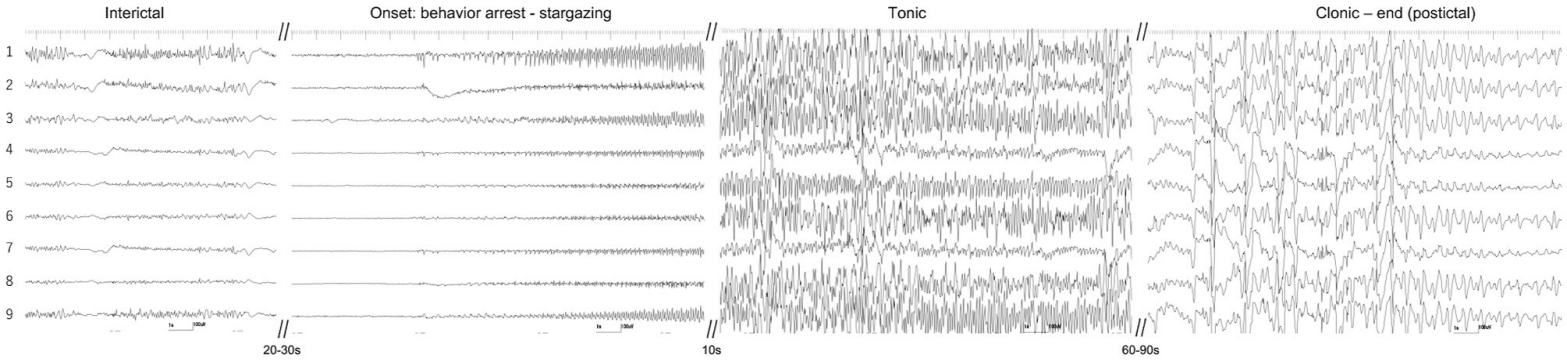
Ictal ECoG of a spontaneous focal limbic seizure evolving into a generalized tonic-clonic seizure. Interictal spikes were mainly observed at electrodes #1 and 9. Seizure activity began as a low-voltage fast wave in all electrodes and the rhythmic discharge started from electrodes #1, 3, and 9. It evolved into a generalized tonic-clonic seizure. See also the movie of intracranial video-EEG (Supplementary Video 1).

Unfortunately, at the initiation of iVEEG monitoring, disconnection of the depth EEGs was noted. Therefore, iVEEG recording was limited to ECoG of the right temporo-occipital area. On interictal ECoG, interictal spikes were dominantly observed on electrodes #1, 3, and 9. During the monitoring period, we observed three spontaneous focal seizures evolving into GTCS. These three seizures and their EEG patterns were very similar. Typically, the cat suddenly stopped his behavior and/or gazed upward with low-voltage and high-frequency activity (suppression background activity) on all ECoG electrodes for approximately 20 s, and rhythmic (10–13 Hz) epileptiform activity with waxing amplitude started from electrode #1, which spread rapidly (<0.5 s) to the surrounding electrodes. At this time, the cat showed ventroflexion of the head, head turning and circling to the left, and facial twitching with more large amplitude discharges, which developed into GTCS (1–2 min). The amplitude and frequency of the epileptiform activity decreased gradually along with seizure termination; the cat and ECoG recovered to the interictal phase (Figure 6 and Supplementary Video 1). We speculated that the first low-voltage and high-frequency activity on ECoG (arresting behavior and star-gazing) represented subcortical seizure activity, that is, hippocampal seizure, and the activity spread to the temporal cortical area, finally developing into a generalized seizure.

### Identification of the Suspected Epileptogenic Zone and Surgical Strategy

Five conceptual abnormal zones (9), that is, symptomatogenic, irritative (EEG abnormal), seizure-onset, structurally abnormal, and functional deficit zones, all of which were suspected from the findings of each preoperative examination mentioned above, are summarized in Table 1. These results allowed us to identify the suspected epileptogenic zone in the right atrophic hippocampus and temporo-occipital cortex underlying ECoG electrodes #1, 3, and 9. According to the feline brain atlas (30), the temporo-occipital cortex showing interictal epileptiform discharges (electrodes #1, 3, and 9) is distributed in the lateral part of the ectosylvian and ectomarginal gyri.

**Table 1:** Suspected epileptogenic zone of the present case.

**Abnormal zone**	**Location**	**Modality**
Symptomatogenic	Hippocampus	Ictal movie
Irritative (EEG abnormal)	Right temporal (T4), occipital (O2), and left occipital (O1)	Interictal scalp EEG
Seizure-onset	Hippocampus to right temporo-occipital cortex	Ictal video EEG (iVEEG monitoring)
Structurally abnormal	Right atrophic hippocampus and temporo-occipital cortex	Structural MRI
Functional deficit	Right forebrain, bilateral occipital lobes	Interictal neurological examination

*EEG, electroencephalography; iVEEG, intracranial video-electroencephalography; MRI, magnetic resonance imaging*.

Although our presurgical evaluations of the cat did not detect the epileptogenic zone as a single focus, it was suspected in the right atrophic hippocampus and/or temporo-occipital cortex. According to human epilepsy surgery strategy (8), if the epileptogenic zone of this cat was present in the right atrophic hippocampus, “MTLE” would be indicated and hippocampectomy would be applied. If the epileptogenic zone presented in the right atrophic temporo-occipital cortex, indicating “extra-temporal or temporal neocortical (lateral temporal lobe) epilepsy,” the cat would be a candidate for focal cortical resection. If both, then both resection surgeries would be needed. Therefore, we planned a two-step surgical approach, that is, cortical resection of the temporo-occipital cortex at first, and then hippocampectomy if the seizures could not be controlled.

### The 1st Surgery: Focal Cortical Resection

At first, we planned to remove the focal temporo-occipital cortex to observe whether cortical resection alone would suppress the seizures. On the 19th day after the electrode-placing surgery, the cat was anesthetized and administered analgesics as in the previous operation. Besides, levetiracetam (20 mg/kg, IV) was administered preoperatively.

After aseptic treatment, the right temporal muscle was detached gently and the disconnected depth electrodes were removed from the brain (the thin leads had broken at the joint of the stainless electrodes). Then, the position of each ECoG electrode on the dura overlying the temporo-occipital region was pictured and the electrodes were removed. Using a surgical microscope (OPMI 6-CH; Carl Zeiss Meditec, Tokyo, Japan), a U-shaped dural incision was made, and a part of the temporo-occipital cortex was exposed. Matching the picture of the position of ECoG electrodes, focal cortical resection was performed carefully to aspirate only gray matter and leave white matter with bipolar cautery and suction (Supplementary Video 2). However, due to the atrophic and thin cortex, some white matter was also removed. The dura, temporal muscle, and skin were sutured. Then, the cat was awakened. Levetiracetam (20 mg/kg, TID, PO) was administered for 1 week postoperatively, and maintenance ASDs (phenobarbital and diazepam) were given continuously. Analgesics and antibiotics were also used postoperatively as in the previous surgical procedure.

### Seizure Outcome After the 1st Surgery

**Figure 7:**
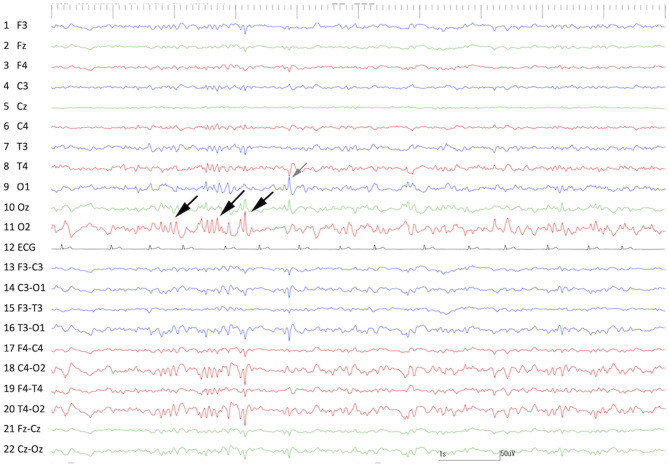
Interictal scalp EEG at 30 days after the 1st surgery (cortical resection). Frequent spikes and polyspikes were observed at O2 (black arrows) and less frequent spikes were also recognized at O1 (gray arrow).

Although the primary neurological deficits (right forebrain signs, bilateral blindness, and cognitive dysfunction) were unchanged, there were no significant postoperative adverse effects or complications. Moreover, for 66 days after surgery, seizure freedom was achieved under maintenance ASD therapy (phenobarbital 2 mg/kg, q12h and diazepam 0.7 mg/kg, q12h). At 1 month (30 days) after surgery, scalp EEG and MRI were performed. On scalp EEG, frequent spikes and polyspikes were still observed on the right occipital region (O2), and less frequent spikes were also observed on the left occipital region (O1) (Figure 7). There was no significant change around the resected cortex on MRI. However, at 67 days after surgery, focal seizures evolving into GTCS were clustered (3 sz/d), which were similar to those observed preoperatively, so it was considered that the seizures also originated from the remaining cortex and/or atrophic hippocampus. Therefore, we prepared for the second operation as initially planned. Finally, two more seizures were counted; therefore, a total of five seizures were observed between the first and second surgeries (5 sz/85 days, 1.8 sz/m on average).

### The Second Surgery: Hippocampectomy and Additional Cortical Resection

At 86 days after the first surgery, the second surgery, consisting of additional cortical resection and total hippocampectomy of the right side, was performed. Anesthesia and presurgical and postsurgical medications were the same as in the first surgery.

**Figure 8:**
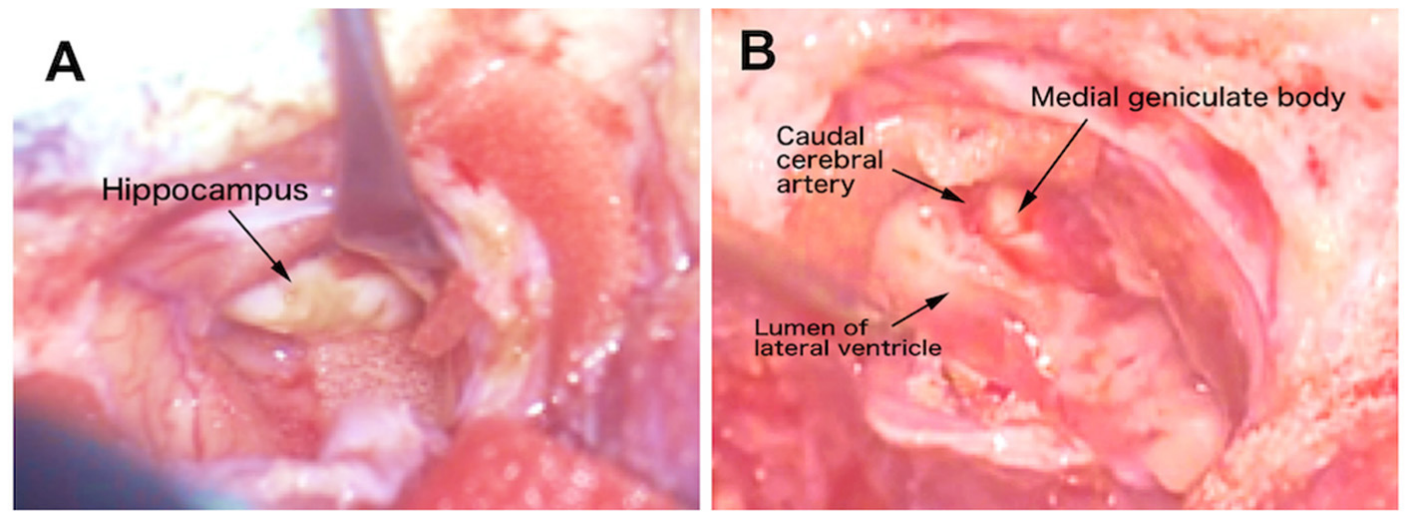
Video captures of the intraoperative microsurgical operation site. **(A)** The appearance of the atrophic hippocampus. **(B)** After hippocampectomy. The caudal cerebral artery and medial geniculate body were seen medially. See also the operation movie shown in Supplementary Video 3.

After the skin incision, by detaching the temporal muscle and dural incision while paying attention to adhesions, the previous operation site, that is, partially resected temporo-occipital cortex, was explored. The site of the resected cortex was covered by pale and spiderweb-like pia and arachnoid maters. After the abruption of those tissues, the underlying white matter was observed. By removing the white matter with suction, the extended lateral ventricle was opened, and the atrophic, slightly wrinkled, and faded hippocampus was observed (Figure 8A and Supplementary Video 3). As the hippocampus is a banana-shaped longitudinal structure extending dorsomedial to ventromedial along the inner aspect of the lateral ventricle, it was divided at the middle, and the dorsal and ventral portions were removed separately.

Initially, the ventral part was slightly rotated medially to visualize the hippocampal head. The border between the hippocampus and amygdala was unclear; therefore, the tip of the hippocampal formation, that is, hippocampal tuberculum, was cauterized. Then, the middle site of the hippocampal body was dissected by bipolar cautery, avoiding the choroid plexus. The ventral portion of the hippocampus was rotated laterally and the medial aspect, including fimbria, was detached from the inner arachnoid, choroid plexus, and blood vessels, and the ventral portion was removed from the lateral ventricle (Supplementary Video 3).

The dorsal end of the hippocampus was dissected by bipolar cautery proximally to the midline, that is, the dentate gyrus tubercle, and the dorsal part of the hippocampal body was peeled away from the medial structures along the fimbria and finally removed from the lateral ventricle (Supplementary Video 3). While the dorsal part was being removed, the dorsolateral aspect of the medial geniculate body and caudal cerebral artery were observed medially (Figure 8B).

After intraventricular handling, intraoperative ECoG was recorded on the residual temporo-occipital cortex to confirm the remaining spikes, and additional resections were performed. The resected cortex and hippocampus were submitted for histopathological evaluation. Finally, the intracranial surgical site was covered with artificial dura (GORE-TEX^®^ ePTFE patch; W.L. Gore & Associates, Newark, DE, USA) after repeated confirmation of hemostasis. It was sealed with factor XII and fibrinogen adhesion (Beriplast P combi-set tissue adhesion; CSL Behring, Tokyo, Japan). The temporal muscle and scalp were closed as usual. The cat recovered without any problems, and postoperative levetiracetam was administered for 1 week and maintenance ASDs were continued as in the postoperative management of the first surgery.

### Seizure Outcome After the Second Surgery

**Figure 9:**
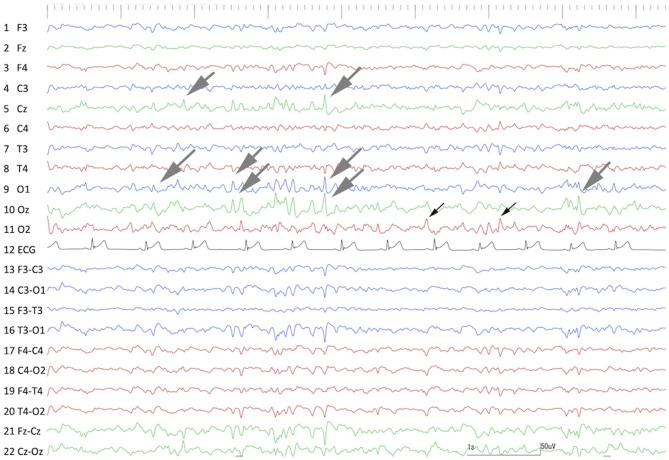
Interictal scalp EEG at 60 days after the 2nd surgery (hippocampectomy). Frequent spikes and polyspikes were observed at O1, Oz, and Cz (gray arrows). Less frequent spikes were still observed at the operated region (O2) (black arrows). Compare to Figure 1.

**Figure 10:**
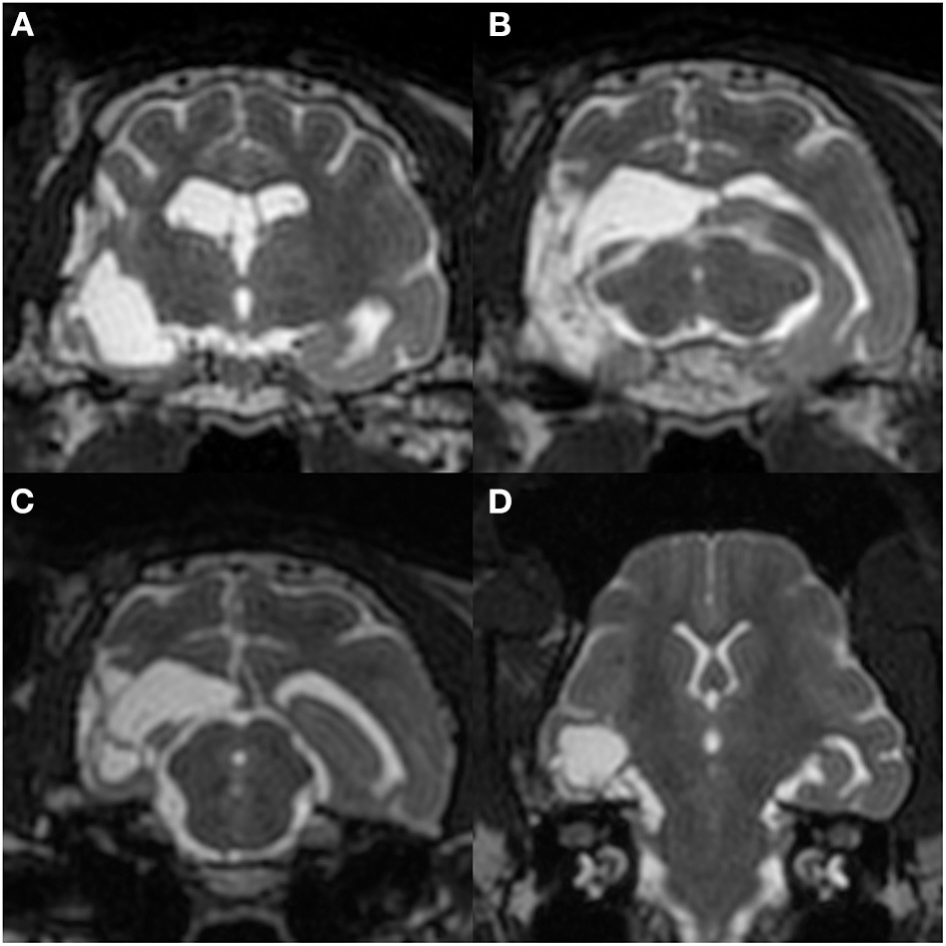
Postoperative MRI at 60 days after the 2nd surgery. Transverse T2-weighted images at the hippocampal head **(A)**, medial geniculate body (**B**; matching Figure 2), and rostral colliculus **(C)**, and a dorsal image perpendicular to the long axis of the hippocampus **(D)**. The right hippocampus and a part of the temporo-occipital cortex had been removed without apparent surrounding signal changes.

As in the first surgery, there were no adverse effects or complications after the second surgery, and neurological status was unchanged from the preoperative state. After the second surgery, no seizures were observed for 91 days; however, frequent spikes on O1 and Oz (left and center of the occipital region) and sporadic spikes in O2 (occipital region on the operated side) were still observed on postoperative scalp EEG, which was performed at 60 days after the second surgery (Figure 9). Concurrent MRI revealed complete resection of the right hippocampus and partially resected temporo-occipital cortex (Figure 10). At 92 days after the second surgery, facial myoclonus with mydriasis followed by GTCS, which had been recognized as a minor seizure type preoperatively, occurred. The same type of seizure was observed four times (at 122, 150, 270, and 401 days after the second surgery) until the cat was euthanized at 1.5 years (597 days) after the second surgery due to the reason described below. Therefore, the final seizure frequency was 0.25 sz/m (<1 sz/3 m) on average, that is, a >85% reduction compared with the preoperative seizure frequency, although ASD therapy (phenobarbital 2 mg/kg, q12h and diazepam 0.7 mg/kg, q12h) had been continued.

At ~1 year and 5 months after the second surgery, the cat developed subacute decreased appetite and spontaneous water intake, leading to dehydration and hypothermia, which required continuous fluid therapy, forced feeding, and warming. Clinicopathological examinations, including complete blood count and serum chemistry, thoracic X-ray, and abdominal ultrasound, were conducted, but they were unremarkable. Despite palliative treatment, the cat gradually weakened and developed to be bedridden and lethargy, resulting in a severe dermal burn by the electric warmer. Finally, the cat was euthanized at 597 days after the second surgery, and necropsy and histopathology of the whole body were performed.

### Histopathology

**Figure 11:**
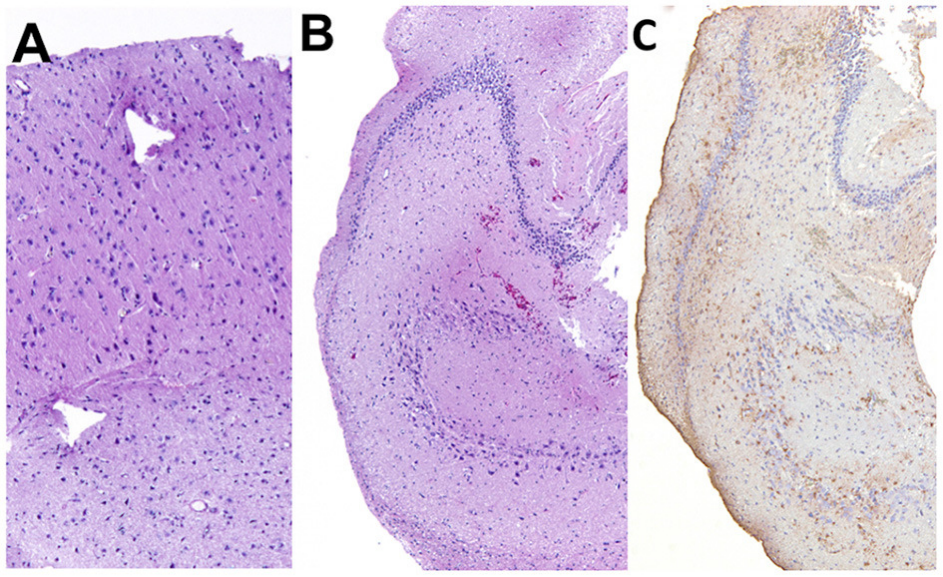
Histopathology of the resected cortex **(A)** and hippocampus **(B,C)**. Shrunken neurons with pyknosis were generally observed in both specimens. In the hippocampus, mild gliosis was observed. **(A,B)** Hematoxylin and eosin staining. **(C)** Anti-glial fibrillary acidic protein immunohistochemistry.

A few parts of the resected lateral temporal cortex and hippocampus were submitted for histopathological evaluations. As the samples were surgically resected tissues, it was difficult to make an appropriate sectional angle for the correct evaluation of the cortex and hippocampus. On the resected cortical and hippocampal specimens, ischemic changes, that is, neuronal shrinking with pyknosis, were observed (Figures 11A,B). However, two board-certified veterinary pathologists judged that these changes were acute or artifactual changes by surgical removal or fixation rather than epileptic changes. Although it was difficult to evaluate the severity of neuronal loss due to the angle of the section and the whole hippocampal structure could not be evaluated, astrocytic glial proliferation was observed in the hippocampus by anti-glial fibrillary acidic protein immunohistochemistry (Figure 11C). A minor hemorrhage and slight infiltration of inflammatory cells around small vessels were also observed in the hippocampus. Other epileptogenic or pathological changes such as focal cortical dysplasia were not observed within the resected sample.

Histopathology of the whole brain collected at necropsy revealed the complete loss of the right hippocampus and a part of the temporo-occipital cortex and the presence of dural adhesions and granulomatous inflammation on the operated site. Additionally, old necrotic lesions with minimum gliosis were observed along the tracts of the depth electrodes. No changes related to epilepsy or death were found in other brain regions. In addition, no evidential findings suggesting the cause of the atrophic changes in the right hippocampus and temporo-occipital cortex were observed; therefore, a congenital or perinatal disorder, such as a focal vascular event, that affected the development of those areas was speculated.

In organs other than the brain, severe suppurative dermatitis, mild interstitial nephritis, chronic cystitis with mild pyelitis, mild lymphocytic cholangitis, and mild extramedullary hematopoiesis in the spleen were observed. Excepting severe suppurative dermatitis, which was the direct cause of euthanasia, there was no lesion that might be related to the cause of dehydration and hypothermia at the end of life.

## Discussion

This is the first exploratory case study of intracranial epilepsy surgery with electroclinical and imaging data in modern veterinary medicine. In the present report, although complete freedom from seizures was not achieved, we demonstrated that epilepsy surgery is feasible in a feline patient with epilepsy and could be an alternative therapy for veterinary patients with DRE.

To date, research reports of epilepsy surgery for dogs or cats are limited to experimental studies for corpus callosotomy on normal dogs (12) or for amygdalohippocampectomy on a feline limbic seizure model induced with kainic acid (31), and experimental studies (14, 32) or a clinical trial (13) of neurostimulation (vagus nerve stimulation and deep brain stimulation) in dogs. However, a few, but very precious, case reports have been published in the veterinary field (18–21, 33).

To our knowledge, the oldest report was published by Oliver in 1965 (19). In this report, an 11-year-old boxer with frequent clonic seizures was operated on without any evidence of the epileptogenic focus. This dog had multiple necrotic lesions on the right occipital and frontal cortexes (i.e., structural epilepsy) that were suctioned, and seizures did not recur for several weeks. However, seizures recurred severely, and the dog died at ~4 months after surgery. Although a histological evaluation was not performed, this was the first report of surgery aimed to prevent seizures in veterinary medicine. In 1971, Parker and Cunningham also reported surgical removal of an epileptogenic focus detected by 8-channel scalp EEG in a dog with structural epilepsy originating from fungal meningoencephalitis (18). In this case, a lateral transfrontal craniotomy was performed to approach the left frontal area, which showed frequent spikes on EEG. The epileptogenic lesion on the surface of the frontal lobe was a mass that was resected and diagnosed histologically as a “mycelioma” of fungal hyphae. Medications for infection were applied continuously, and this case survived for approximately 3 months after surgery without any seizures; however, it died unexpectedly from an unknown cause. This case report proved the utility of scalp EEG to detect an epileptogenic focus, and the lesion was found in the location indicated by the EEG findings. However, at that time, presurgical information using neuroimaging, which would reveal the characteristics, extension, or multiple occurrences of lesions, was not available. After that, as the use of advanced imaging modalities such as computed tomography and MRI has spread in the veterinary field, intracranial surgery for structural lesions that may cause recurrent seizures has become common for mass lesions. In contrast, surgery combined with electrodiagnostic evidence has not been performed (20, 21, 33).

In the present case, the seizure semiology observed from the first presentation until before surgery was consistent with FTLE, which was characterized by focal limbic seizures consisting of arrest, staring, facial twitching, and mydriasis, then contralateral head turning, circling, and hemi-limb clonus, and finally evolving into GTCS (22, 24, 25). These signs, especially for the first observed behavioral arrest, that is, staring and facial twitching, suggested that the symptomatogenic zone was located in the hippocampus or amygdala-hippocampus complex. In addition to seizure semiology, hippocampal sclerosis, atrophy, or necrosis is a well-known imaging and pathological change as a cause or result of TLE in humans and cats (6–8, 26, 29, 34–36). Although the MRI and histological findings of the resected hippocampus in this case did not correspond to typical hippocampal sclerosis and the hippocampal abnormality might have been congenital, there was an apparent loss of right hippocampal volume on structural MRI (volumetry and voxel-based morphometry) and gliosis was confirmed histologically. These imaging and pathological results, that is, structurally abnormal zone, and seizure outcome, as well as the fact that the seizure signs had changed postoperatively, suggested that the right atrophic hippocampus, at least, was included in the epileptogenic zone. Similar to the hippocampus, the right temporo-occipital cortex also showed atrophy and ischemic changes; however, cortical resection of these area performed in the first operation did not prevent seizures, even though seizure freedom was achieved for 2 months. Therefore, we thought retrospectively that, as also described below, this atrophic cortex was not the true or primary epileptogenic zone.

It is considered that chronic iEEG, that is, ECoG and depth EEG, which is direct recording from the cortex, with video monitoring (iVEEG), which allows us to observe seizure symptoms, is the best approach to suggest the true epileptogenic zone [6, 7, 19, 33]. However, iEEG requires invasive procedures and its recording site is limited to a small area, which makes it possible to miss the true seizure-onset zone. In fact, we placed a 9-channel ECoG grid on the atrophic temporo-occipital cortex in the present case, and ictal ECoG showed low-amplitude and high-frequency activity at the onset of seizure symptoms (behavioral arrest; early phase), which then gradually became rhythmic discharges from specific electrodes (#1, 3, and 9) with increasing amplitude as the seizure symptoms changed (drop and head turning, hemi-clonic, and GTCS; middle to late phase) (Figure 6 and Supplementary Video 1). As the depth electrodes inserted into the hippocampus were broken before monitoring, we had recorded ECoG only and decided to resect the focal cortex showing ECoG abnormalities at the first surgery. As a result, the same seizure type as observed before surgery reoccurred; namely, we could not determine the true epileptogenic zone from ECoG. The observed low-amplitude and high-frequency activity at the early phase is well-known in human and experimental epilepsy (37–39). This EEG pattern is classified as a type 1 or 3 seizure pattern, which is most commonly observed in human patients with hippocampal-onset TLE (37, 39). This low-amplitude high-frequency activity on ictal iEEG is considered an indicator of the seizure-onset zone, although its mechanism of generation is still controversial (38). However, in current epileptology, this concept of low-amplitude high-frequency activity is incorporated into the concept of “high frequency oscillations,” which are recognized as a specific biomarker of an epileptogenic zone (38, 40). Unfortunately, because the EEG system we used could not record the wide frequency band, we could not assess high-frequency oscillations. In addition, if the depth electrodes had not been broken and could record hippocampal EEG or if we placed the ECoG grid more ventromedially to cover the pyriform cortex, we might have been able to detect the true epileptogenic zone, that is, the hippocampus, and to perform hippocampectomy in a single surgery.

Regarding the functional deficit zone in the present case, the right forebrain dysfunction and bilateral cortical blindness were recognized by routine neurological examinations and were consistent with the structural MRI lesions, except for the left visual (occipital) cortex, which did not have an apparent lesion. To detect the functional deficit zone related to the seizure-onset zone more accurately, functional imaging such as diffusion and perfusion MRI, EEG-functional MRI, and nuclear imaging would be essential (9). However, in this case, we could only obtain interictal DWI, which was not informative. We tried to identify interictal perfusion MRI and/or interictal-early postictal changes using diffusion and perfusion MRI preoperatively, which is suggested to be useful for the detection of the functional deficit zone in feline epilepsy (27, 28). Still, we failed due to various factors such as the failure of a bolus injection of contrast agent or missing the timing of the scanning. Similar to the abovementioned depth-EEG, if we could have performed those techniques, the functional deficit zone might have been detected more accurately. Conversely, the dysfunction of the left occipital cortex (blindness of the right eye) was MRI-negative. However, this site showed EEG abnormalities, even though they were less frequent than in the right hemisphere, and might be another epileptogenic focus for the seizures that remained after the second surgery, as discussed later.

The present case was diagnosed as drug-resistant FTLE with atypical atrophy of the hippocampus and temporo-occipital cortex, although the cause of that was suspected to be a congenital or perinatal disorder. In human medicine, MTLE is the most common form of DRE and most patients with MTLE are suitable for epilepsy surgery (7, 8, 41–43). Many studies, including systematic reviews, meta-analyses, and randomized controlled studies, have shown that surgical treatment for MTLE is superior to medical management and 60–80% patients achieve postoperative seizure freedom (41–43). In human patients with MTLE and hippocampal sclerosis, the rate of seizure freedom is increased to 80–90% (44). Although we could not determine whether the present case had typical hippocampal sclerosis, the right hippocampus was apparently atrophic, and its surgical removal resulted in a remarkable reduction of seizures. In human medicine, there are two main surgical methods for resecting mesial temporal structures, that is, anterior temporal lobectomy and selective amygdalohippocampectomy *via* several different approaches; however, there is no statistically significant difference in seizure outcome between these techniques (42). Anterior temporal lobectomy and selective amygdalohippocampectomy in human MTLE both involve hippocampal resection; however, it does not represent a total resection, but a resection of <1.5 cm from the tip of the hippocampal head for the dominant hemisphere or <3 cm for the non-dominant side. Conversely, there is no report regarding surgical resection of the hippocampus in veterinary medicine. However, the approach to temporal lobectomy for hemangioma removal at the level of the hippocampal head in one dog has been reported (20). Experimentally, selective amygdalohippocampectomy (resection of the amygdala and ventral portion of the hippocampus) has been reported in a chronic feline epilepsy model with kainic acid microinjection into the unilateral amygdala (31). This study reported that selective amygdalohippocampectomy could inhibit seizures from the original site (unilateral kainate-injected amygdala); however, seizures from the contralateral amygdala and/or hippocampus occurred at 2 weeks after surgery. Furthermore, there was no detailed description of behavioral or clinical signs and postoperative complications in that report. In the present case, we performed total hippocampectomy with a partial cortical resection of the temporo-occipital region, but the cat did not show any additional behavioral or neurological changes or complications. There is a possibility that we could not notice subtle neurological or functional changes because the present case already had chronic neurological deficits including cognitive dysfunction, blindness, and right forebrain signs before surgery. In human studies, the complications of anterior temporal lobectomy or selective amygdalohippocampectomy with or without preoperative iVEEG monitoring are relatively rare (~5%) (45). However, they include memory impairment (especially in the dominant hemisphere), dysphasia or speech error (in the dominant hemisphere), contralateral visual field defect, depression, ipsilateral cranial neuropathy (mainly for cranial nerves III or IV), and hemiplegia or hemiparesis, in addition to surgical complications such as infection, meningitis, cerebrospinal fluid leak, hemorrhage, hematoma, vasospasm and stroke, and death (7, 8, 41, 45). To date, there has been no report regarding the technique and complications for hippocampectomy in veterinary patients.

Notably, Zilli et al. reported a cadaveric study of cortico-hippocampectomy in non-epileptic cats to develop epilepsy surgery for FTLE (17). The surgical procedure described in that study was highly similar to that performed in the present case; however, the extent of the surgical field in the present case was more widespread than that in the cadaveric study. Therefore, both papers suggest that the ectosylvian gyrus for cortical incision and visual confirmation of the caudal cerebral artery and lateral aspect of the medial geniculate body after hippocampal removement are surgical landmarks when approaching and removing the feline hippocampus, respectively. In the non-epileptic cadaveric study, the authors could not remove the hippocampus totally, especially because of the difficulty to remove part of its head and tail, while we performed total hippocampectomy in the present case. As the reason for this, in the present case, the hippocampus and lateral cortex were extremely atrophied, resulting in an extended lateral ventricle, which made it easy for us to maneuver within the lateral ventricle for hippocampal resection. Therefore, the difficulty/ease of total hippocampectomy (with or without removal of the amygdala) may depend on the space of the surgical field in each case. In addition, it is speculated that a wider cortical resection and/or different approaches are needed in a case with normal hippocampal and lateral ventricular size. Conversely, another argument is whether total resection of the hippocampus is required to achieve seizure control in feline patients with DRE. In fact, hippocampectomy in anterior temporal lobectomy and selective amygdalohippocampectomy in humans is limited to the hippocampal head, as mentioned above. In order to discuss the procedure and efficacy of hippocampectomy in veterinary patients with epilepsy, further experimental studies and the accumulation of clinical cases are needed.

As mentioned above, surgery for MTLE in human medicine results in a good outcome as mentioned above (41–43). Seizure outcome following epilepsy surgery is commonly evaluated according to an outcome classification system. The most widely used system is “Engel's classification,” which was suggested by Engel in 1992, but a new classification has recently been suggested by the International League Against Epilepsy (ILAE) (Table 2) (46). In the present case, postoperative seizure frequency (4 sz/year) was improved significantly (80–90% reduction) from the preoperative baseline (2–3 sz/m). When judged with the above human classification systems, the surgical seizure outcome of the present case could be classified as Engel Class IIIA and ILAE Class 4. However, in our case, we consider that the recurrent seizures observed after surgery were the minor seizure type from the contralateral occipital lobe that had been observed since the cat was 10 years of age (described below), while the original (major) type was inhibited by surgery. Therefore, if the classification systems were adopted to the original seizure type only, the outcome would correspond to Engel Class IA or ILAE Class 1.

**Table 2:** Classification systems of surgical seizure outcome.

	**Engel's Classification (1992)**	**ILAE Classification (2001)**
I (1)	**Free of disabling seizures**	
	A: Completely seizure-free since surgery	Completely seizure-free: no aura
	B: Non-disabling simple partial seizures only since surgery	
	C: Some disabling seizures after surgery, but free of disabling seizures for at least 2 years	
	D: Generalized convulsion with ASD discontinuation only	
II (2)	**Rare disabling seizures (“almost seizure free”)**	
	A: Initially free of disabling seizures, but now has rare seizures	Only aura; no other seizure
	B: Rare disabling seizures since surgery	
	C: More rare disabling seizures since surgery, but rare seizures for the last 2 years	
	D: Nocturnal seizures only	
III (3)	Worthwhile improvement	1–3 seizure days per year; ± aura
	A: Worthwhile seizure reduction^⋆^	
	B: Prolonged seizure-free intervals amounting to greater than half the followed-up period, but not <2 years	
IV (4)	**No worthwhile improvement**	
	A: Significant seizure reduction	4 seizure days per year to 50% reduction of baseline seizure days^⋆^; ± aura
	B: No appreciable change	
	C: Worse seizures	
V (5)		<50% reduction of baseline seizure days to 100% increase of baseline seizure days; ± aura
VI (6)		>100% increase of baseline seizure days; ± aura

⋆ *Classification of the present case. ILAE, international league against epilepsy*.

Regarding the postoperative seizures, we considered that they originated from the left occipital lobe, that is, the mirror focus against the original (the right) temporo-occipital cortex, although we could neither detect those minor seizures during the iVEEG monitoring period nor place ECoG electrodes on the left hemisphere. Due to our delayed decision and preparations for epilepsy surgery, these minor but remaining seizures may have been produced by secondary epileptogenesis by the long-term duration of DRE from the original (primary) focus. This point is crucial in determining the timing of epilepsy surgery, especially for focal epilepsy. Although there is reportedly no significant relationship between the duration of epilepsy and postoperative seizure outcome in human TLE, there is a slight tendency that patients with a shorter duration have a better outcome than those with a longer duration (47). However, the accumulated evidence suggests that early surgical therapy is recommended for patients with DRE, especially with structural epilepsy such as MTLE with hippocampal sclerosis, cavernous malformations, or a benign tumor (44, 48–50). Furthermore, long-term unilateral TLE reportedly induces bilateral epileptogenicity (secondary epileptogenesis) and an unfavorable postsurgical outcome (51). Therefore, if we could have performed epilepsy surgery on the present case at a much earlier stage, for example, before the age of 10 years when the cat exhibited the minor seizure type, it might have been possible to inhibit secondary epileptogenicity and provide a better postoperative seizure outcome.

In conclusion, we reported our experience of resection epilepsy surgery and its 1.5-year follow-up in a feline case with drug-resistant structural epilepsy. Although some recurrent seizures that might have originated from the hemisphere contralateral to the surgical side remained after surgery, focal cortical resection and hippocampectomy of the presumed primary epileptogenic zone by presurgical evaluations resulted in a remarkable reduction of seizure frequency without further complications. Although additional basic and clinical studies for presurgical evaluations, techniques for epilepsy surgery, and other ways to treat feline DRE are needed, this case report shows the possibility that intracranial epilepsy surgery with advanced neuromodalities may be a treatment option for DRE in veterinary medicine.

## Data Availability Statement

The original contributions presented in the study are included in the article/Supplementary Material, further inquiries can be directed to the corresponding author/s.

## Ethics Statement

The animal study was reviewed and approved by The Institutional Animal Care and Use Committee and Animal and Human Biology Research Ethics Committee of the Nippon Veterinary and Life Science University (accession numbers; 27S-60, 28K-6, 29K-2, and 2019K-1).

## Author Contributions

DH, RA, YH, YY, TK, and SM: conception, design, and data acquisition. DH, RA, YH, JC, and KU: analysis and interpretation of data and preparing figures. DH and YY: drafting the manuscript. All authors: revising and approval of the final manuscript.

## Conflict of Interest

The authors declare that the research was conducted in the absence of any commercial or financial relationships that could be construed as a potential conflict of interest.

## Supplementary Material

Supplementary Data 1 Materials and methods for EEG, MRI, intracranial electrodes and epilepsy monitoring cage.

Supplementary Video 1 Intracranial video-EEG of a focal limbic seizure evolving into a generalized tonic-clonic seizure.

Supplementary Video 2 Movie of the first surgery: focal cortical resection in the ectosylvian gyrus.

Supplementary Video 3 Movie of the second surgery: hippocampectomy.
