# Corrigendum: Focal Cortical Resection and Hippocampectomy in a Cat With Drug-Resistant Structural Epilepsy

**DOI:** 10.3389/fvets.2021.744820

**Published:** 2021-08-19

**Authors:** Daisuke Hasegawa, Rikako Asada, Yuji Hamamoto, Yoshihiko Yu, Takayuki Kuwabara, Shunta Mizoguchi, James K. Chambers, Kazuyuki Uchida

**Affiliations:** ^1^Laboratory of Veterinary Radiology, Faculty of Veterinary Science, Nippon Veterinary and Life Science University, Musashino, Japan; ^2^The Research Center for Animal Life Science, Nippon Veterinary and Life Science University, Musashino, Japan; ^3^Veterinary Medical Teaching Hospital, Nippon Veterinary and Life Science University, Musashino, Japan; ^4^Laboratory of Veterinary Pathology, Graduate School of Agricultural and Life Sciences, The University of Tokyo, Bunkyo-ku, Japan

**Keywords:** cat, drug-resistant epilepsy, electroencephalography, electrocoricography, epileptogenic zone, epilepsy surgery, magnetic resonance imaging, video-EEG

In the original article, there was a mistake in Figure 4 as published. The left and right sides of the middle and right colored apparent diffusion coefficient maps of the lower row in the original article were reversed. The corrected revised Figure 4 appears below.

**Figure 4:**
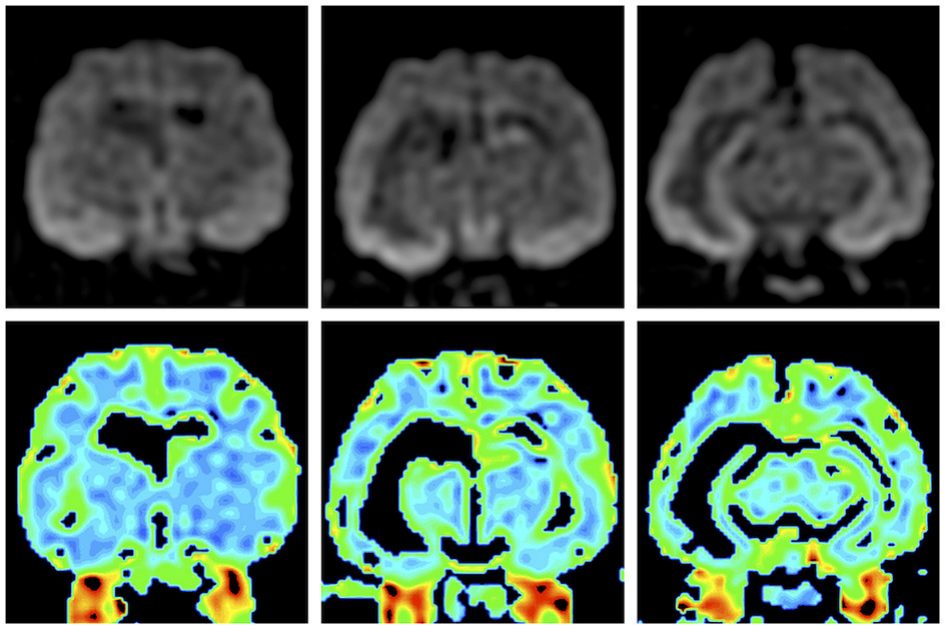
Preoperative isotropic diffusion-weighted imaging (upper row) and colored apparent diffusion coefficient maps (lower row). Bilateral hippocampi and the right amygdala and temporo-occipital cortex showed hyperintensity, but there were no significant changes on the apparent diffusion coefficient map.

In the published article, there was an error in affiliation 2. Instead of “Research Center of Animal Life Science”, it should be “Research Center for Animal Life Science.”

In the published article, the title of Dr. Masaki Iwasaki was missing. His title is “MD, Ph.D.”

The authors apologize for this error and state that this does not change the scientific conclusions of the article in any way. The original article has been updated.

## Publisher's Note

All claims expressed in this article are solely those of the authors and do not necessarily represent those of their affiliated organizations, or those of the publisher, the editors and the reviewers. Any product that may be evaluated in this article, or claim that may be made by its manufacturer, is not guaranteed or endorsed by the publisher.

