# Corrigendum: Focal Cortical Resection and Hippocampectomy in a Cat With Drug-Resistant Structural Epilepsy

**DOI:** 10.3389/fvets.2021.760886

**Published:** 2021-09-15

**Authors:** Daisuke Hasegawa, Rikako Asada, Yuji Hamamoto, Yoshihiko Yu, Takayuki Kuwabara, Shunta Mizoguchi, James K. Chambers, Kazuyuki Uchida

**Affiliations:** ^1^Laboratory of Veterinary Radiology, Faculty of Veterinary Science, Nippon Veterinary and Life Science University, Musashino, Japan; ^2^The Research Center for Animal Life Science, Nippon Veterinary and Life Science University, Musashino, Japan; ^3^Veterinary Medical Teaching Hospital, Nippon Veterinary and Life Science University, Musashino, Japan; ^4^Laboratory of Veterinary Pathology, Graduate School of Agricultural and Life Sciences, The University of Tokyo, Bunkyo-ku, Japan

**Keywords:** cat, drug-resistant epilepsy, electroencephalography, electrocoricography, epileptogenic zone, epilepsy surgery, magnetic resonance imaging, video-EEG

In the original article, there was an error, the sex of the case cat was incorrect.

A correction has been made to Abstract and Case Presentation, and Long-Term iVEEG monitoring sections. The term “female” has been corrected to “male,” and also “her” was changed into “his,” respectively.

In the original article, the names of the authors were spelt incorrectly in reference 11. Michael G, Jones C, Mary R, Packer A, Royal T, Volk HA, et al. should be Jones GMC, Volk HA and Packer RMA. The corrected reference appears below.

Jones GMC, Volk HA, Packer RMA. Research priorities for idiopathic epilepsy in dogs : viewpoints of owners, general practice veterinarians, and neurology specialists. *J Vet Intern Med*. (2021) 35:1–14. doi: 10.1111/jvim.16144

The authors apologize for these errors and state that this does not change the scientific conclusions of the article in any way. The original article has been updated.

## Publisher's Note

All claims expressed in this article are solely those of the authors and do not necessarily represent those of their affiliated organizations, or those of the publisher, the editors and the reviewers. Any product that may be evaluated in this article, or claim that may be made by its manufacturer, is not guaranteed or endorsed by the publisher.

