# Supplementary material for: Focal Cortical Resection and Hippocampectomy in a Cat With Drug-Resistant Structural Epilepsy
Source: Front Vet Sci. 2021 Jul 20;8:719455. doi: 10.3389/fvets.2021.719455 (PMC8329420; doi:10.3389/fvets.2021.719455)
Supplement: Supplementary Data 1 — Materials and methods for EEG, MRI, intracranial electrodes and epilepsy monitoring cage. [file Data_Sheet_1.DOCX]

Supplementary Data Sheet

# Scalp EEG

Preoperative and postoperative (follow-up) scalp EEGs were recorded for approximately 30 min with a digital EEG system (Neurofax EEG-1200; Nihon Kohden, Tokyo, Japan) under sedation with medetomidine (20 µg/kg, IM, Domitor; Zenoaq, Tokyo, Japan). The recording conditions were as follows: sampling frequency = 1,000 Hz, high-cut filter = 60 Hz, time constant = 0.1, and AC filter = on. Body temperature was maintained at 37–38 ºC using hot-water bags. Recording subdermal needle electrodes were placed on the frontal (F3, F4), central (parietal) (C3, C4), temporal (T3, T4), and occipital (O1, O2) regions bilaterally (odd = left; even = right) and on 3 midline points (Fz, Cz, Oz). Electrocardiograms were also recorded with the EEG system. Although montage (i.e., remontage) could be changed suitably in the recorded EEG, reviewing montages were referential (monopolar; reference was average referential or digital zero) and longitudinal bipolar derivations, as shown in Figures 1, 7, and 9 in the main text. EEG was reviewed on the EEG viewer of the internal software in the EEG system by one board-certified veterinary neurologist (DH) at a tracing speed of 10–15 s/view.

# MRI sequences

Preoperative and postoperative (follow-up) MRI was performed with a 3.0 Tesla superconducting MRI system (Signa HDxt; GE Healthcare, Tokyo, Japan). General anesthesia was induced by propofol (7 mg/kg, IV, 1% Propofol injection; Maruishi Pharmaceutical, Osaka, Japan) and maintained by isoflurane inhalation (Isoflu; DS Pharma Animal Health, Osaka, Japan) and oxygen. During anesthesia, lactated Ringer’s fluid was infused at 3 mL/kg/h. Body temperature was kept between 37–38 ºC with a blanket and/or hot-water bags. The cat was positioned in a sternal recumbent position on the table, and its head was placed in a human knee 8-channel array RF coil. The following sequences were acquired: 3D-T2 cube (FSE, TR/TE = 3,200/78.5 ms, FOV = 15 × 15 cm, slice thickness = 0.6 mm, matrix = 256 × 256, NEX = 1), 3D-T1 SPGR (SPGR, TR/TE/TI = 6.5/3.1/450 ms, FOV = 15 × 15 cm, matrix = 256 × 192, NEX = 1) with and without gadodiamide (0.1 mmol/kg, IV, Omniscan; GE Healthcare), transverse T2-weighted (FSE, TR/TE = 7,000/85 ms, FOV = 15 × 15 cm, slice thickness = 2.0 mm, slice gap = 0.5 mm, matrix = 384 × 288, NEX = 1), T2-fluid-attenuated inversion recovery (FSE, TR/TE/TI = 11,000/140/2,400 ms, FOV = 15 × 15 cm, slice thickness = 2.0 mm, slice gap = 0.5 mm, matrix = 256 × 192, NEX = 2), T2*-weighted (GRE, TR/TE = 740/18 ms, FOV = 15 × 15 cm, slice thickness = 3.0 mm, slice gap = 0.5 mm, matrix = 320 × 192, NEX = 2), DWI (FSE propeller, TR/TE = 8,000/72 ms, b = 1,000, 3 axes, FOV = 15 × 15 cm, slice thickness = 2.0 mm, slice gap = 0.5 mm, matrix = 128 × 128, NEX = 1), and diffusion tensor imaging (EPI, TR/TE = 8,000/94 ms, b = 1,000, 15 axes, FOV = 15 × 15 cm, slice thickness = 2.4 mm, slice gap = 0.5 mm, matrix = 128 × 128, NEX = 2). Apparent diffusion coefficient and fractional anisotropy values were calculated and mapped by the internal software of the MRI system.

Bilateral hippocampi were traced semi-manually and their volumes were measured from 3D-T2 cube data using the imaging work station (Virtual Place; Fujin AZE, Tokyo, Japan) as described previously (Ref. 26 in the main text). Voxel-based morphometry analysis (comparing the case with 12 healthy control cats) was performed using 3D-T1 data and previously produced feline standard template and tissue probability maps (Ref. 29 in the main text).

# Intracranial electrodes placement

An overview of the intracranial electrode placement procedure is provided in the main manuscript. Herein, we describe the more detailed procedures.

## Insertion and stabilization of deep electrodes

Stereotaxic coordinates of the bilateral amygdala and ventral hippocampus were measured from the CT-MRI Fusion images acquired preoperatively. The stereotaxically stabilized brain has a zero plane in each of the three axes; the anterior (A)-posterior (P) direction = transverse plane, the horizontal (H) direction = dorsal plane, and the left (L)-right (R) direction = sagittal plane). The transverse zero (A=0) plane is the level of the external auditory meatus where the bilateral ear bars are inserted; the dorsal zero (H=0) plane connects the left and right ear bars with the eye bars fixed at the infraorbital margin of the zygomatic bone on each side; and the zero plane in the left-right direction (L/R=0) is the midline. However, the horizontal direction was measured as the depth (D) from the brain surface and skull surface because the depth from the brain surface rather than the coordinates from the zero plane is more practical when inserting the electrode. After the measurements, we confirmed that there was no considerable discrepancy with the corresponding coordinates in the stereotaxic atlas of the cat brain (Snider and Niemer. A stereotaxic atlas of the cat brain. University of Chicago Press, 1961). Finally, the coordinates of the amygdala and hippocampus of this case were as follows: the left and right amygdala = A 12 mm, L/R 10 mm, D 19 mm from the brain surface (24 mm from the bone surface); the left ventral hippocampus = A 8 mm, L 13 mm, D 18 (22) mm; the right ventral hippocampus = A 6 mm, R 10.5 mm, D 18.7 (23) mm. After making a bar hole in the skull on each coordinate, the electrode manipulator attached to the stereotaxic instrument was used to insert a deep electrode into each target coordinate. After the electrode was inserted, the electrode was stabilized by filling the bar hole with dental resin. After stabilization of the electrode, the electrode was removed from the electrode manipulator (Fig. 5A).

## Placement and stabilization of ECoG electrodes

After craniectomy of the right temporal bone, ECoG electrodes were placed on the dura in the temporal-occipital region. In human medicine, ECoG electrodes are generally placed subdurally and covered by the dura mater. However, in cats, it is challenging to place ECoG electrodes subdurally due to the extremely thin and easily torn dura. And also, ECoG in this species can be recorded adequately with the epidural electrodes. A groove was made in the skull at the position where the electrode lead would run so that the electrode would fit well over the dura mater (Fig. 5A). An artificial dura was placed over the exposed area of the brain where the ECoG electrode was placed. The temporal muscle was sutured to the bone at the edge of the craniectomy area so that it covered the dura. The lead wire was fixed by suturing to the temporal muscle.

# Epilepsy monitoring cage

Continuous iVEEG was monitored within an epilepsy monitoring cage for 18 days. The inner dimensions of the cage were 70 cm height × 60 cm width × 70 cm depth; five sides, except the front, were made of stainless steel, and the front door was made of acrylic for video monitoring. A 15-channel 360º-rotating slip-ring connector (NSR-15; Neuroscience Inc., Tokyo, Japan) that allowed wired EEG recording in a freely moving animal was attached to the ceiling of the cage.

In the previously reported feline (and/or rodent) experimental studies using freely moving (V)EEG monitoring, intracranial electrodes were usually connected to a socket, which was fixed on the skull with dental cement (Refs. 25 and 31 in the main text). However, we thought such an experimental method should not be applied to clinical veterinary cases due to problems of infection, appearance, and ethics. Therefore, referring to the methodology in human VEEG monitoring, we tunneled the lead wires subcutaneously and derived them from the nape. Those lines were connected to the slip-ring connector via a relay cable long enough for the cat to move freely in the monitoring cage. The wound through which the electrode wires emerged and the connector between the electrode wires and relay cable were wrapped with gauze and fastened to the neck with an adhesive bandage. The wound was treated with gentamicin ointment daily until no serous fluid was produced, after which the bandage was changed every 3 days. Recorded iVEEG data were stored in the internal hard disk of the EEG system. The recording conditions were the same as used for scalp EEG.
